# Cost-Effectiveness of Frontline Treatment for Advanced Renal Cell Carcinoma in the Era of Immunotherapies

**DOI:** 10.3389/fphar.2021.718014

**Published:** 2021-09-09

**Authors:** SiNi Li, JianHe Li, LiuBao Peng, YaMin Li, XiaoMin Wan

**Affiliations:** ^1^ Clinical Nursing Teaching and Research Section, The Second Xiangya Hospital, Central South University, Changsha, China; ^2^ The Xiangya Nursing School, Central South University, Changsha, China; ^3^ Department of Pharmacy, The Second Xiangya Hospital, Central South University, Changsha, China

**Keywords:** cost-effectiveness, renal cell carcinoma, microsimulation, immune checkpoint inhibitors, immunotherapy

## Abstract

**Background:** Recent randomized controlled trials have demonstrated that immune checkpoint inhibitors (ICIs) improve patient outcomes, but whether these novel agents are cost-effective for untreated advanced renal cell carcinoma (aRCC) remains unclear.

**Materials and Methods:** A microsimulation model was created to project the healthcare costs and outcomes of six strategies (lenvatinib-plus-pembrolizumab, nivolumab-plus-cabozantinib, nivolumab-plus-ipilimumab, pembrolizumab-plus-axitinib, avelumab-plus-axitinib, and sunitinib monotherapy) for patients with aRCC. Transition probability of patients was estimated from CLEAR, CheckMate 9ER, CheckMate 214, KEYNOTE-426, JAVELIN Renal 101, and other data sets by using parametric survival modeling. Lifetime direct medical costs, life years (LYs), quality-adjusted LYs (QALYs), and incremental cost-effectiveness ratios (ICERs) were estimated from a United States payer perspective. One-way and probabilistic sensitivity analyses were performed, along with multiple scenario analyses, to evaluate model uncertainty.

**Results:** Of the six competing strategies, nivolumab-plus-cabozantinib yielded the most significant health outcomes, and the sunitinib strategy was the least expensive option. The cost-effective frontier consisted of the nivolumab-plus-cabozantinib, pembrolizumab-plus-axitinib, and sunitinib strategies, which displayed the ordered ICERs of $81282/QALY for pembrolizumab-plus-axitinib vs sunitinib and $453391/QALY for nivolumab-plus-cabozantinib vs pembrolizumab-plus-axitinib. The rest of the strategies, such as lenvatinib-plus-pembrolizumab, nivolumab-plus-ipilimumab, and avelumab-plus-axitinib, were dominated. The cost of sunitinib drove the model most influentially.

**Conclusions:** For aRCC, the pembrolizumab-plus-axitinib strategy is likely to be the most cost-effective alternative at the willingness-to-pay threshold of $100,000.

## Introduction

Renal cell carcinoma (RCC) is the most common type of kidney cancer, with more than 73,000 cases diagnosed and 14,000 deaths in 2020 in the United States (Choueiri et al., 2015; National Cancer Institute, 2021). Advanced RCC (aRCC) accounts for the highest death rate among kidney cancers because this disease is usually asymptomatic at the initial stage; the 5-year relative survival rate for aRCC is only 11% (Fisher et al., 2013; Bhatt and Finelli, 2014; Amzal et al., 2017; Sarfaty et al., 2018).

Sunitinib, once the mainstay target drug for the treatment of aRCC, has been substituted by novel immune checkpoint inhibitor (ICI) agents on the basis of survival data reported in multiple previous studies. Recently, CheckMate 9ER, a large open-label phase three trial, compared nivolumab combined with cabozantinib with sunitinib in treatment-naïve patients with aRCC (Choueiri et al., 2021). In this study, nivolumab-plus-cabozantinib was associated with significantly longer overall survival (OS) and progression-free survival (PFS) than was sunitinib (Choueiri et al., 2021). The median PFS in the nivolumab-plus-cabozantinib arm vs. sunitinib arm was 16.6 vs. 8.3 months (hazard ratio [HR], 0.51; 95% confidence interval [CI], 0.41–0.64). The proportion of patients with 12-month OS was 85.7% with the nivolumab-plus-cabozantinib strategy vs. 75.6% with sunitinib strategy (Choueiri et al., 2021). Another randomized phase three trial (CLEAR) revealed that lenvatinib-plus-pembrolizumab showed significant improvement when compared with sunitinib with respect to OS (median, 14.7 vs. 9.2 months; HR, 0.65; 95% CI, 0.53–0.80) and PFS (median, 23.9 vs. 9.2 months; HR, 0.39; 95% CI, 0.32–0.49) (Motzer et al., 2021). Moreover, multiple randomized controlled trials (RCTs) have reported that compared with sunitinib, ICI-based regimens (including nivolumab-plus-ipilimumab, pembrolizumab-plus-axitinib, and avelumab-plus-axitinib) can enhance survival and quality of life (QoL) (Motzer et al., 2018; Motzer et al., 2019; Powles et al., 2020).

Although ICIs have obviously improved health outcomes in patients with aRCC, it is still unclear whether the substantial ICI cost and adverse events (AEs) are justified by the health benefits gained, the decreased health resource consumption of subsequent line of treatments, or both. Under the current healthcare setting, patients, physicians, and policy makers alike need reasonable evidence as a framework to determine the value of different agents in oncology. Therefore, the aim of this study was to estimate the cost-effectiveness of ICI treatments compared with each other and with sunitinib as a first-line treatment for aRCC from a United States payer perspective, using the most recently reported RCT data.

## Methods

### Analytic Overview

**FIGURE 1:**
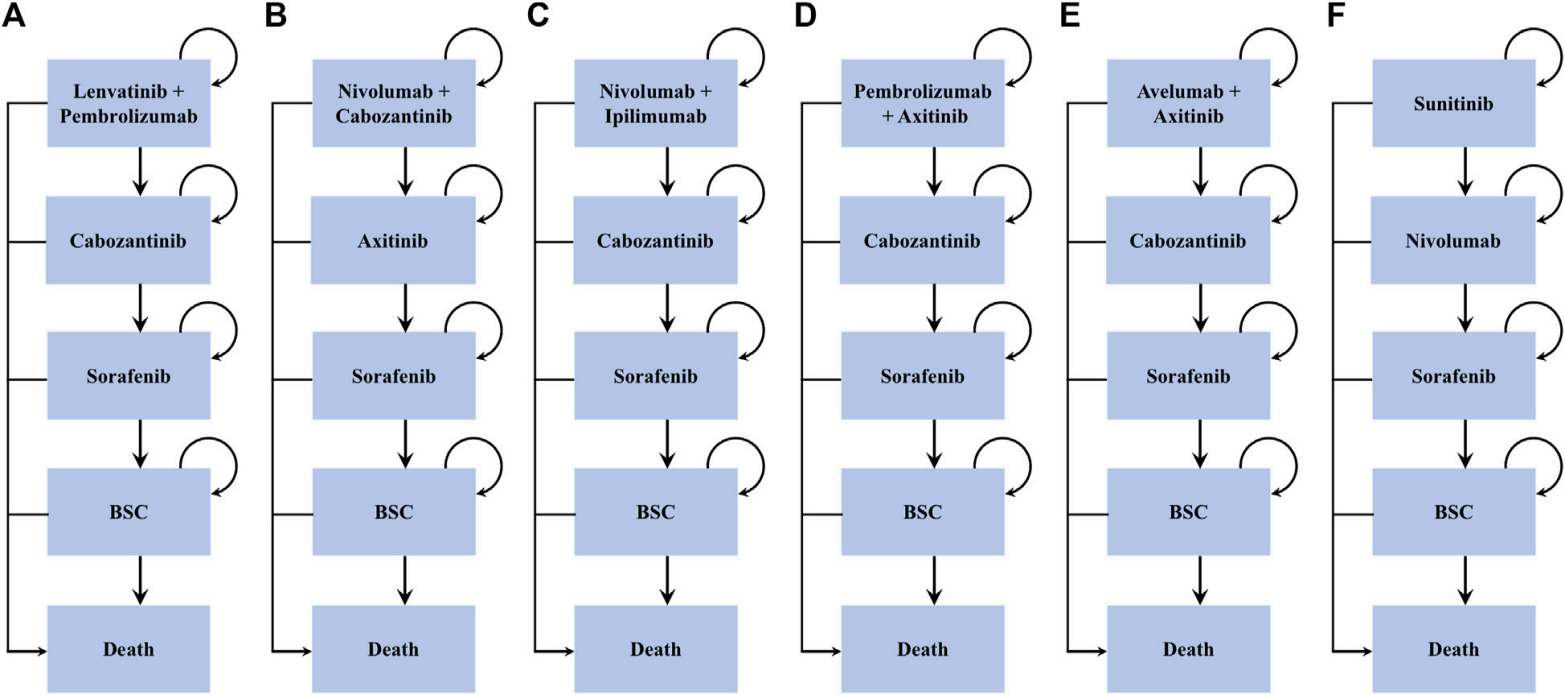
Treatment sequences. *BSC = best support care. Arrows represent patients staying on current treatment or moving to the next line of treatment.

A microsimulation model was developed to evaluate the lifetime health and economic outcomes of six treatment strategies for patients with treatment-naïve aRCC by using TreeAge Pro (TreeAge Software, Williamstown, MA, United States) (Figure 1 and Supplementary Figure S1). The baseline sample of patients was generated to mirror the respective RCTs (Motzer et al., 2018; Motzer et al., 2019; Powles et al., 2020; Choueiri et al., 2021; Motzer et al., 2021) (Supplementary Table S1). The mean age of the patients was 62 years (obtained by averaging the ages of the patients in the six RCTs), and all individuals had clear cell type aRCC (Motzer et al., 2018; Motzer et al., 2019; Powles et al., 2020; Choueiri et al., 2021; Motzer et al., 2021).

Individuals entered the model and received one of the six frontline interventions: 1) lenvatinib-plus-pembrolizumab, 2) nivolumab-plus-cabozantinib, 3) nivolumab-plus-ipilimumab, 4) pembrolizumab-plus-axitinib, 5) avelumab-plus-axitinib, and 6) sunitinib. Patients who progressed to lenvatinib-plus-pembrolizumab, nivolumab-plus-ipilimumab, and pembrolizumab-plus-axitinib subsequently received cabozantinib as the second-line treatment, while those progressing to nivolumab-plus-cabozantinib and sunitinib subsequently received axitinib and nivolumab, respectively. Sorafenib was administered as the third-line therapy among all arms. All patients who progressed following sorafenib were switched to the best supportive care (BSC) phase before death (Figure 1). All dosage and administration schedules for each line of therapy were collected from the respective RCTs and are displayed in Supplementary Table S2 (Rini et al., 2011; Motzer et al., 2013; Motzer et al., 2014; Motzer et al., 2015; Choueiri et al., 2016; Motzer et al., 2018; Motzer et al., 2019; Powles et al., 2020; Rini et al., 2020; Choueiri et al., 2021; Motzer et al., 2021).

The model cycle was 42 days, and lifetime horizon was used to estimate the total costs, life years (LYs), quality-adjusted LYs (QALYs), and incremental cost-effectiveness ratios (ICERs). All the costs were adjusted to 2021 USD, and both cost and outcomes were discounted by 3% annually (Weinstein et al., 1996). This cost-effectiveness analysis was performed from a United States payer perspective with a willingness-to-pay threshold (WTP) of $100,000/QALY (Neumann et al., 2014).

### Transition Probability

Based on transition probabilities estimated from the survival curves of the respective RCTs, patients transitioned among different health states (Rini et al., 2011; Motzer et al., 2013; Motzer et al., 2014; Motzer et al., 2015; Choueiri et al., 2016; Motzer et al., 2018; Motzer et al., 2019; Powles et al., 2020; Rini et al., 2020; Choueiri et al., 2021; Motzer et al., 2021). On the basis of the PFS Kaplan–Meier curves from the RCTs (Rini et al., 2011; Motzer et al., 2013; Motzer et al., 2014; Motzer et al., 2015; Choueiri et al., 2016; Motzer et al., 2018; Motzer et al., 2019; Powles et al., 2020; Rini et al., 2020; Choueiri et al., 2021; Motzer et al., 2021), the probability of patients remaining in the PFS state of each strategy was estimated by using the standard extrapolation technique derived by Guyot et al. (2012). In brief, the survival data of PFS derived from the Kaplan–Meier curves (Rini et al., 2011; Motzer et al., 2013; Motzer et al., 2014; Motzer et al., 2015; Choueiri et al., 2016; Motzer et al., 2018; Motzer et al., 2019; Powles et al., 2020; Rini et al., 2020; Choueiri et al., 2021; Motzer et al., 2021) were extracted to generate pseudo individual patient-level data. Then, these reconstructed survival data were fit to four standard parametric models (exponential, Weibull, lognormal, and log-logistic), and the suitable survival distribution selected for each PFS curve based on the goodness of fit (Akaike information criterion). The PFS data of the sunitinib strategy in the five trials (Motzer et al., 2018; Motzer et al., 2019; Powles et al., 2020; Choueiri et al., 2021; Motzer et al., 2021) and sorafenib in the two trials (Motzer et al., 2014; Rini et al., 2020) were pooled, given the comparable trial eligibility criteria and patient baseline characteristics (Supplementary Table S1) among the RCTs, similar to the analyses by Wu and Shi (2020).

This model also took into consideration discontinuation of treatment associated with AEs, with transition probabilities collected from literature (Rini et al., 2011; Motzer et al., 2013; Motzer et al., 2014; Motzer et al., 2015; Choueiri et al., 2016; Motzer et al., 2018; Motzer et al., 2019; Powles et al., 2020; Rini et al., 2020; Choueiri et al., 2021; Motzer et al., 2021). Finally, the overall transition probability for death during each line of active treatment was calculated by combining an age-specified background mortality rate from the 2017 United States Life Table (Arias et al., 2019) with the data concerning treatment-related severe AEs from the RCTs (Rini et al., 2011; Motzer et al., 2013; Motzer et al., 2014; Motzer et al., 2015; Choueiri et al., 2016; Motzer et al., 2018; Motzer et al., 2019; Powles et al., 2020; Rini et al., 2020; Choueiri et al., 2021; Motzer et al., 2021). The probability of death from the BSC state was estimated on the basis of the OS curve of the RECORD-1 trial using the same approach as with the transition probabilities of PFS (Motzer et al., 2008). Baseline evaluates for the clinical transition probabilities are displayed in Table 1.

**TABLE 1:** Input parameters.

Parameters	Mean	Range	Distribution	References
Survival model of PFS in the full cohort				
Lenvatinib + pembrolizumab	Shape =1.176426		Weibull	Motzer et al. (2021)
	Scale = 28.71649			
Nivolumab + cabozantinib	Shape = 1.569		Loglogistic	Choueiri et al. (2021)
	Scale = 15.064			
Nivolumab + ipilimumab	Shape = 1.2291		Loglogistic	Motzer et al. (2018)
	Scale = 12.2534			
Pembrolizumab + axitinib	Shape = 1.328		Loglogistic	Powles et al. (2020)
	Scale = 16.108			
Avelumab + axitinib	Shape = 1.1701		Loglogistic	Motzer et al. (2019)
	Scale = 12.8368			
Sunitinib	Shape = 1.4257		Loglogistic	Motzer et al. (2018); Motzer et al. (2019); Powles et al. (2020); Choueiri et al. (2021); Motzer et al. (2021)
	Scale = 9.3505			
Cabozantinib	Shape = 1.824		Loglogistic	Choueiri et al. (2016)
	Scale = 7.476			
Nivolumab	Shape = 1.5169		Loglogistic	Motzer et al. (2015)
	Scale = 5.0424			
Axitinib	Shape = 1.4633		Loglogistic	Rini et al. (2011); Motzer et al. (2013)
	Scale = 6.6318			
Sorafenib	Shape = 2.281		Exponential	Motzer et al. (2014); Rini et al. (2020)
OS in the best support care	Shape = 1.613		Loglogistic	Motzer et al. (2008)
	Scale = 13.857			
Probability of treatment discontinuation as a result of AE (%)				
Lenvatinib + pembrolizumab	37.2		Beta	Motzer et al. (2021)
Nivolumab + cabozantinib	19.7		Beta	Choueiri et al. (2021)
Nivolumab + ipilimumab	22.0		Beta	Motzer et al. (2018)
Pembrolizumab + axitinib	48.0		Beta	Powles et al. (2020)
Avelumab + axitinib	7.6		Beta	Motzer et al. (2019)
Sunitinib	13.74		Beta	Motzer et al. (2018); Motzer et al. (2019); Powles et al. (2020); Choueiri et al. (2021); Motzer et al. (2021)
Cabozantinib	12.0		Beta	Choueiri et al. (2016)
Nivolumab	8.0		Beta	Motzer et al. (2015)
Axitinib	8.49		Beta	Rini et al. (2011); Motzer et al. (2013)
Sorafenib	18.11		Beta	Motzer et al. (2014); Rini et al. (2020)
Probability of treatment mortality as a result of AE (%)				
Lenvatinib + pembrolizumab	3.13		Beta	Motzer et al. (2021)
Nivolumab + cabozantinib	0.31		Beta	Choueiri et al. (2021)
Nivolumab + ipilimumab	1.46		Beta	Motzer et al. (2018)
Pembrolizumab + axitinib	4.00		Beta	Powles et al. (2020)
Avelumab + axitinib	0.7		Beta	Motzer et al. (2019)
Sunitinib	1.27		Beta	Motzer et al. (2018); Motzer et al. (2019); Powles et al. (2020); Choueiri et al. (2021); Motzer et al. (2021)
Cabozantinib	8.00		Beta	Choueiri et al. (2016)
Nivolumab	0		–	Motzer et al. (2015)
Axitinib	0		–	Rini et al. (2011); Motzer et al. (2013)
Sorafenib	0.7		Beta	Motzer et al. (2014); Rini et al. (2020)
Probability of background death	–	–	–	Arias et al. (2019)
Drug cost				
Lenvatinib 20 mg	379.702	303.76–455.64	Gamma	CMS (2021)
Pembrolizumab 200 mg	10129.60	8103.68–12155.52	Gamma	Su et al. (2021)
Nivolumab 240 mg	6849.84	5479.87–8219.81	Gamma	Su et al. (2021)
Ipilimumab 10 mg	1572.25	8980.70–13471.04	Gamma	Su et al. (2021)
Axitinib 5 mg	265.05	212.04–318.06	Gamma	Watson et al. (2020)
Avelumab 10 mg	85.331	4874.10–7311.16	Gamma	Su et al. (2021)
Sunitinib 50 mg	623.08	498.46–747.70	Gamma	Lu et al. (2020)
Cabozantinib 60 mg	491.30	393.04–589.56	Gamma	Lu et al. (2020)
Sorafenib 200 mg	174	139.20–208.80	Gamma	CMS (2021)
Cost of BSC	1,256	1,022–1,489	Gamma	Lu et al. (2020)
Management of AEs				
Lenvatinib + pembrolizumab	598.20	478.56–717.84	Gamma	Perrin et al. (2015); Wan et al. (2019); Lu et al. (2020); Agency for Healthcare Research and Quality, US Dept of Health and Human Services (2021); Centers for Medicare and Medicaid Services (2021); Motzer et al. (2021)
Nivolumab + cabozantinib	1214.68	971.74–1457.61	Gamma	Perrin et al. (2015); Swallow et al. (2018); Wan et al. (2019); Lu et al. (2020); Choueiri et al. (2021)
Nivolumab + ipilimumab	2862.07	2289.66–3434.48	Gamma	Motzer et al. (2018); Watson et al. (2020); Agency for Healthcare Research and Quality, US Dept of Health and Human Services (2021)
Pembrolizumab + axitinib	3393.27	2714.61–4071.92	Gamma	Powles et al. (2020); Watson et al. (2020); Agency for Healthcare Research and Quality, US Dept of Health and Human Services (2021)
Avelumab + axitinib	3840.88	3072.70–4609.06	Gamma	Motzer et al. (2019); Bensimon et al. (2020); Agency for Healthcare Research and Quality, US Dept of Health and Human Services (2021)
Sunitinib	6632.78	5306.22–7959.34	Gamma	Motzer et al. (2018); Motzer et al. (2019); Bensimon et al. (2020); Powles et al. (2020); Agency for Healthcare Research and Quality, US Dept of Health and Human Services (2021); Choueiri et al. (2021); Motzer et al. (2021)
Cabozantinib	5188.85	4151.08–6226.62	Gamma	Choueiri et al. (2016); Bensimon et al. (2020); Agency for Healthcare Research and Quality, US Dept of Health and Human Services (2021)
Nivolumab	534.45	427.56–641.34	Gamma	Swallow et al. (2018)
Axitinib	4660.34	3728.27–5592.41	Gamma	Rini et al. (2011); Motzer et al. (2013); Swallow et al. (2018)
Sorafenib	2284.81	556.72–835.08	Gamma	Motzer et al. (2014); Perrin et al. (2015); Wan et al. (2019); Lu et al. (2020); Rini et al. (2020); Agency for Healthcare Research and Quality, US Dept of Health and Human Services (2021)
Administration cost				
IV infusion, single or initial drug (≤1 h)	148.3	118.64–177.93	Gamma	Centers for Medicare and Medicaid Services (2021)
IV infusion, each sequential drug (≤1 h)	71.88	57.504–86.256	Gamma	Centers for Medicare and Medicaid Services (2021)
Utilities				
First-line treatment	0.82	0.65–0.98	Beta	Wan et al. (2019)
Second-line treatment	0.77 (SD: 0.24)	0.616–0.924	Beta	Cella et al. (2018)
Third-line treatment	0.66 (SD: 0.30)	0.528–0.792	Beta	de Groot et al. (2018)
Fourth-line treatment, BSC	0.494	0.403–0.570	Beta	Patel et al. (2021)
Disutility due to AEs (grade ≥3)	0.157	0.11–0.204	Beta	Wu et al. (2018)
Average patient weight (kg)	70	49.0–93.8	Beta	Watson et al. (2020)

OS, overall survival; PFS, progression-free survival; AE, adverse event; BSC, best support care.

### Costs and Utilities

The costs and utilities incorporated in the model are listed in Table 1. Only direct costs were considered, including drugs, administration, management of AEs, and BSC. The unit prices of pembrolizumab, nivolumab, ipilimumab, and avelumab in the United States were estimated based on the 2021 average sale price from the Centers for Medicare and Medicaid Services (CMS) (CMS, 2021). The costs of the oral drugs, including lenvatinib, axitinib, sunitinib, cabozantinib, and sorafenib, were derived from public databases and the literature (CMS, 2021; Su et al., 2021; Lu et al., 2020). Although the mean patient weight in the United States is 74.7 kg, the price of medications was estimated using a patient weight of 70 kg, accounting for weight loss effects in disease (Wan et al., 2019; Lu et al., 2020). The administration fee was based on the 2021 CMS Physician Fee Schedule, with the duration of drug infusion based on RCTs and United States Food and Drug Administration package inserts (Centers for Medicare & Medicaid Services, 2021). The overall costs associated with the management of grade 3 or 4 AEs and BSC were obtained from previous literature (Perrin et al., 2015; Swallow et al., 2018; Wan et al., 2019; Bensimon et al., 2020; Lu et al., 2020; Watson et al., 2020; Agency for Healthcare Research and Quality, US Dept of Health and Human Services (2021)).

The health utility scores, which range from 0 (death) to 1 (perfect health), were collected from published literature (Cella et al., 2018; de Groot et al., 2018; Wan et al., 2019; Patel et al., 2021). In this study, the first-, second-, and third-line treatments and the BSC phases were assigned utility values of 0.82, 0.77, 0.66, and 0.494, respectively. We also adopted a utility decrement (−0.157), specifying the reduction in the valued QoL for AEs (Wu et al., 2018). Based on utility calculations for every health state, QALYs were estimated by weighting patient survival.

### Sensitivity Analyses

Sensitivity analyses, including one-way sensitivity analyses and probability sensitivity analyses (PSA), were incorporated to assess the robustness of the model and test the uncertainty in estimates of variables. In the one-way sensitivity analyses, for parameters with CIs, the upper and lower limits were changed for their 95% CIs; otherwise, the parameters were varied by a 20% change from the base case value to determine their impact on the ICER, in accordance with the existing approach (Goulart and Ramsey, 2011; Zhang et al., 2012; Kohn et al., 2017). During PSA, a Monte Carlo simulation of 2000 iterations of 5,000 patients was generated by using prespecified distributions to sample the critical input parameters. Cost parameters were described by gamma distribution, utility by beta distribution, and the median starting age and weight by normal distribution. On the basis of data from 5,000 iterations, a cost-effectiveness acceptability curve was drawn to illustrate the likelihood that a competing strategy would be regarded as cost-effective at various willingness-to-pay (WTP) threshold levels for health gains (QALYs).

Four scenario analyses were also incorporated in this study. In the first scenario analysis, we varied the time horizon at 5, 10, and 20 years to assess the influence of OS and PFS extrapolations used in the model. In the second, patients would experience a fixed percentage (between 10 and 30%) to elect for BSC after progressing from the first- or second-line treatment instead of receiving next-line treatment. In the third, we prescribed nivolumab or axitinib to replace cabozantinib as the second-line therapy in the model. For the second-line treatments that had progressed after the first-line use of nivolumab (axitinib), the second-line drugs were changed to axitinib (nivolumab). We developed the final scenario analysis accommodating indication-specific pricing, where the cost of nivolumab used in combination with cabozantinib in the first-line treatment varied from the price of nivolumab monotherapy used at second-line setting.

## Results

### Base Case Analysis

Examining six treatment strategies incrementally (Table 2), nivolumab-plus-cabozantinib, pembrolizumab-plus-axitinib, and sunitinib constituted the cost-effective frontier (Supplementary Figure S2). Supplementary Figure S2 illustrates that sunitinib strategy was the least expensive, and compared with this strategy, the pembrolizumab-plus-axitinib strategy gained an extra 0.39 QALYs with an additional cost of $31700.08, which resulted in an ICER of $81282/QALY and dominated the nivolumab-plus-ipilimumab and avelumab-plus-axitinib strategies. The lenvatinib-plus-pembrolizumab strategy was dominated by the nivolumab-plus-cabozantinib strategy, which yielded the greatest health outcomes with an incremental 0.47 QALYs and $213093.73 compared with the pembrolizumab-plus-axitinib strategy. The ICER of the nivolumab-plus-cabozantinib versus pembrolizumab-plus-axitinib strategy was $372109/QALY more than pembrolizumab-plus-axitinib versus sunitinib strategy.

**TABLE 2:** Base case results.

Strategy	Total cost	LY	QALY	ICER^a^
Sunitinib	239257.68	2.99	2.13	Dominated
Avelumab + axitinib	432403.81	3.07	2.32	Dominated
Nivolumab + ipilimumab	306201.03	3.21	2.42	Dominated
Lenvatinib + pembrolizumab	562080.09	3.44	2.61	Dominated
Pembrolizumab + axitinib	270957.76	3.31	2.52	81,282
Nivolumab + cabozantinib	484051.49	3.91	2.99	453391

LY, life year; QALY, quality-adjusted life year; ICER, incremental cost-effectiveness ratio.

a The ICER was compared with the next best nondominated option.

### One-Way Sensitivity and Probability Analyses

**FIGURE 2:**
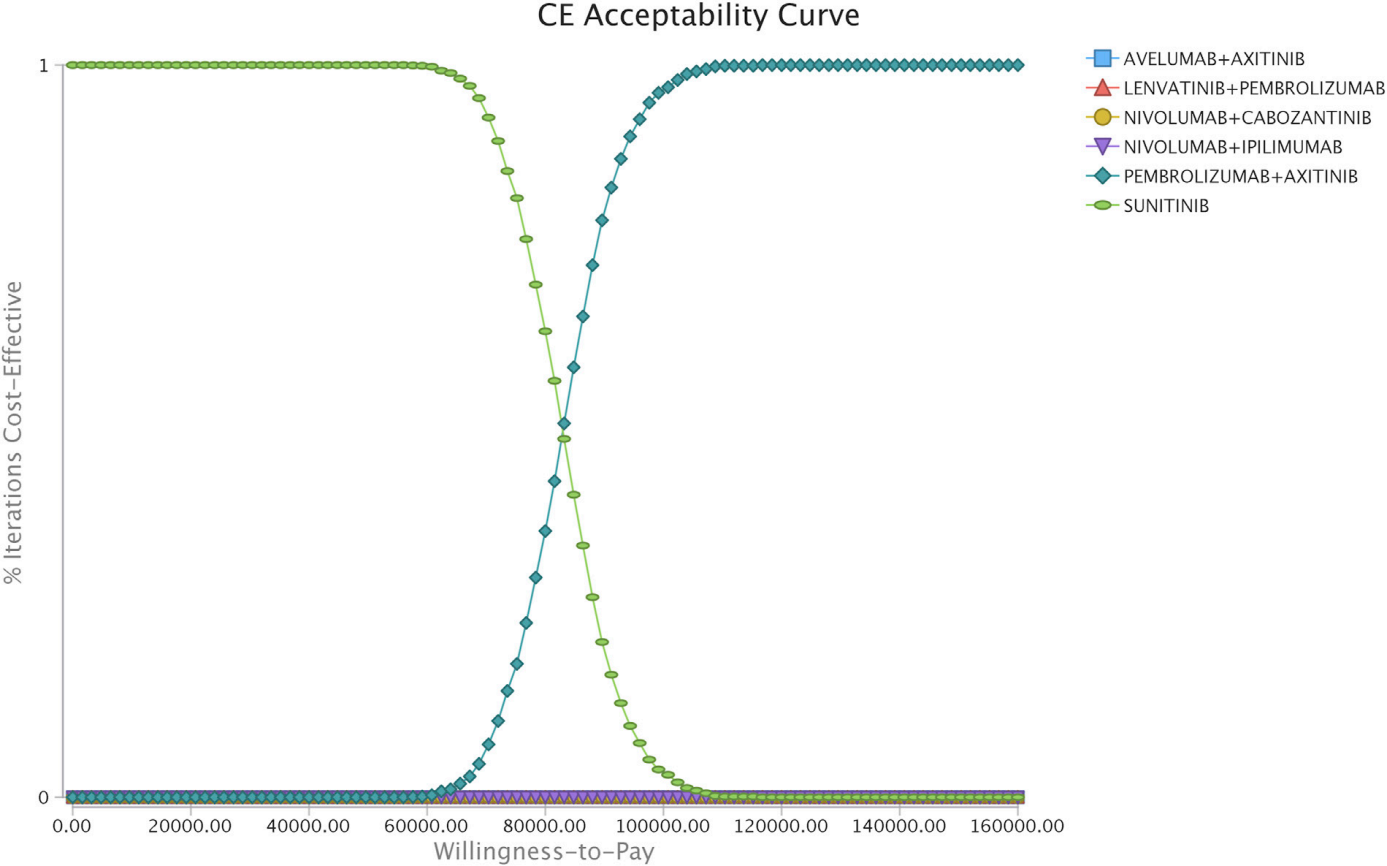
Acceptability curves comparing the cost-effectiveness of different competing strategies in the scenario of the simultaneous competition of six strategies.

In the comparison between the pembrolizumab-plus-axitinib and sunitinib strategies, the one-way sensitivity analyses indicated that the model outcome was considerably impacted by the cost of sunitinib and pembrolizumab, and the weight of patients (Supplementary Figure S3). In the comparison between the nivolumab-plus-cabozantinib and pembrolizumab-plus-axitinib strategies, the age of the patients at the start of treatment, the utility of the second-line of treatment, and cost of nivolumab played a crucial role in the model outcomes (Supplementary Figure S4). Other model input parameters, such as the cost of AE management and discount rate, had a moderate or less influence on the estimated ICER. The cost-effectiveness acceptability curves revealed that the 96 and 100% probabilities of the pembrolizumab-plus-axitinib strategy were considered the cost-effective options at the WTP threshold of $100,000/QALY, when compared with sunitinib (Figure 2).

### Scenario Analyses

The first scenario analyses showed that when the time horizon was adjusted to 5 years, the model produced a higher ICER than the base case analyses because most of the medical costs (95%) were spent in the first 5 years of the time horizon, but patients continued to obtain benefit after 5 years. However, the ICERs were not changed significantly when the time horizon was varied to 10 and 20 years. In the second scenario analyses, a small percentage of patients switched to the BSC phase after disease progression from the first- or second-line therapy rather than receiving next-line treatment. And the results demonstrated that this adjustment did not largely change the model outcomes, with the ICERs for the pembrolizumab-plus-axitinib versus sunitinib strategies being $80186.46/QALY and $82077.16/QALY when modeling 30 and 10% of patients switching to the BSC phase. In the third scenario analyses, nivolumab or axitinib was substituted as the second-line therapy for cabozantinib. The results indicate that the use of nivolumab in the pembrolizumab-plus-axitinib versus sunitinib strategies as a second-line treatment was associated with a more acceptable ICER ($30382.22/QLAY) than in the base case analyses; by contrast, the use of axitinib was associated with a higher ICER of $455856.73/QALY. In the final scenario analyses, reductions in the acquisition prices for nivolumab used in the first-line treatment of 75, 50, and 25% were found to lead to lower ICERs of 259256.70/QALY, $172683.55/QALY, and 83,020.19/QALY, respectively. All the results of the scenario analyses are reported in Supplementary Table S4.

## Discussion

This study suggests that among the six competing strategies, the upfront use of nivolumab-plus-cabozantinib maximized health outcomes, followed by lenvatinib-plus-pembrolizumab and pembrolizumab-plus-axitinib strategies. And as demonstrated in the economic analysis, the nivolumab-plus-cabozantinib, pembrolizumab-plus-axitinib, and sunitinib strategies can be regarded as the most potentially cost-effective options on the frontier line, while the rest of the competing strategies were dominated either because of their lower health benefits and higher costs or not being considered cost-effective as the ICER far exceeded the WTP threshold of the United States. The most influential input parameter driving this model was the cost of sunitinib.

Although there are several cost-effectiveness analyses focusing on ICIs as the first-line of treatment for patients with aRCC, the present study provides the most comprehensive economic evaluation to date that compares different treatment strategies for patients with aRCC. This study also has several potential strengths. First, to the authors' knowledge, this is the first cost-effectiveness study that has incorporated nivolumab-plus-cabozantinib and lenvatinib-plus-pembrolizumab strategies in the first-line setting for patients with aRCC. Second, the majority of parameters incorporated in the model have been based on large, multicenter, randomized, phase 3 clinical trials. Third, contemporary multiagent treatment sequences were predefined to reflect most advances in the treatment of aRCC, and the model presented in this study takes AEs into consideration, including treatment discontinuation due to AEs as well as costs and disutility related to drug toxicity. Forth, a scenario analysis was also performed to reflected the situation in clinical practice in that part of the patients will not receive subsequent treatment due to other causes. Finally, this study was conducted by adopting a microsimulation model to account for the heterogeneity of patients.

Based on the base case analyses, scenario analyses were additionally performed to evaluate drug acquisition prices by implementing indication-specific pricing in the final scenario analysis. And this study demonstrated that considerable price decrement (75–25%) to nivolumab used in the first-line setting resulted in lower ICERs than in base-line outcomes. The results of this study, combined with a series of previous cost-effectiveness studies that have reported high ICERs for aRCC medicines, enhance the requirement for alternative pricing schemes for this disease, such as value-based pricing (Bach and Pearson, 2015) or indication-specific pricing (Bach, 2014). In the United States, although such schemes could decrease consumer surplus and result in profit maximization, the pricing of cancer drugs have minimal association with clinical utility because the United States statutes force its largest insurer (Medicare) to reimburse all approved cancer treatments, restricting negotiations with pharmaceutical companies (Mailankody and Prasad, 2015; Prasad and Mailankody, 2016). By comparison, other countries, such as the regulatory body of the United Kingdom—the National Institute for Health and Care Excellence, that oversee the approval and reimbursement of novel agents on the basis of health economic evaluation (Patel et al., 2021). Therefore, it is crucial to update the policy in the United States that could offer potential to rationally align the drug prices of novel therapeutics with their clinical efficacy, and incentivize the research and development of highly effective treatments (Bach, 2014).

Although this study has important strengths, there are weaknesses that should be considered. First, data were incorporated from several RCTs because of lack of head-to-head data. This indirect comparison presents some biases of the model due to there being some differences among patient characteristics. And although the PFS of the model was validated at each treatment line, the OS curve of the model could not be externally validated because of limited long-term survival data for patients treated with ICIs as the frontier line (Supplementary Figures S5–8). It is necessary to evaluate the concordance of these modeled health outcomes with real-world data and long-term RCTs. Second, modeling the health outcomes of multiagent treatment sequences relies on precise data concerning discontinuation of treatment due to some unrelated reasons for disease progression. Although treatment discontinuation was assessed based on multiple respective RCTs, uncertainty remained regarding the probability of treatment discontinuation in the post-trial period. Third, the health utilities used in this model, although obtained from previously published aRCC cost-effectiveness analyses, may not precisely reflect the hypothetical population simulated in the present study. Accuracy and robustness of model might improve when health utilities estimated for patients with aRCC in populations with Immunotherapy are available in the future. Forth, same as the limitation of other cost-effectiveness analyses, the results of this study cannot be transferred to other countries because the large variation in healthcare systems and policies will result in different health outcomes for different countries (Li et al., 2021). Finally, we did not incorporate a societal perspective because of the theoretical challenge related to gathering the costs and benefits across different sectors and individuals together, including costs related to both informal and non-health sectors.

## Conclusion

In summary, for patients with aRCC, the first-line therapy approaches of the pembrolizumab-plus-axitinib strategy could be regarded as a more cost-effective option for the current WTP threshold of $100,000 in the United States.

## Data Availability

The original contributions presented in the study are included in the article/Supplementary Material; further inquiries can be directed to the corresponding authors.
