# Supplementary material for: Cost-Effectiveness of Frontline Treatment for Advanced Renal Cell Carcinoma in the Era of Immunotherapies
Source: Front Pharmacol. 2021 Sep 9;12:718014. doi: 10.3389/fphar.2021.718014 (PMC8458866; doi:10.3389/fphar.2021.718014)

**eTable 1. Summary table of included studies.**

|  | Key characteristic | | | | | | | | | |
| --- | --- | --- | --- | --- | --- | --- | --- | --- | --- | --- |
| Study | Study design | Treatment | Sample size | Type | Prior therapies | Age (yrs) | Male (%) | IMDC prognostic risk score | | |
|  |  |  |  |  |  |  |  | F | I | P |
| First-line treatment | | | | | | | | | | |
| CLEAR | Phase III RCT | LEN+PEM  LEN+EVE  Sunitinib | 355  357  357 | CC | None | 64  62  61 | 71.8  74.5  77 | 31  31.9  34.7 | 59.2  54.6  53.8 | 9.3  11.8  10.4 |
| CheckMate 9ER | Phase III open-label RCT | NIV+ Cabo  Sunitinib | 323  328 | CC | None | 62  61 | 77.1  70.7 | 22.9  22.0 | 58.2  57.3 | 18.9  20.7 |
| CheckMate 214 | Phase III RCT | NIV+IPI  Suinitinib | 550  546 | CC | None | 62  62 | 75  72 | 23  23 | 61  61 | 17  16 |
| KEYNOTE-426 | Phase III open-label RCT | PEM+AXI  Sunitinib | 432  429 | CC | None | 62  61 | 71  75 | 32  31 | 55  57 | 13  12 |
| JAVELIN Renal  101 | Phase III RCT | AVE+AXI  Sunitinib | 442  444 | CC | None | 62  61 | 71.5  77.5 | 21.3  21.6 | 61.3  62.2 | 16.3  16.0 |
| Second-line treatment | | | | | | | | | | |
| METEOR | Phase III open-label RCT | Cabo  Sunitinib | 331  322 | CC | 1 or more prior  TKI | 63  62 | 77  73 | NA | | |
| CheckMate 025 | Phase III open-label RCT | Nivolumab  Everolimus | 410  411 | CC | 1-2 prior antiangiogenic therapy | 62  62 | 77  74 | NA | | |
| AXIS | Phase III open-label RCT | Axitinib  sorafenib | 361  362 | CC | 1 prior first-line treatment | 61  61 | 73  71 | NA | | |
| Third-line treatment | | | | | | | | | | |
| Robert J Motzer et al., 2014 | Phase III RCT | Dovitinib  Sorafenib | 280  284 | CC | 1 prior TKI plus 1 prior mTORi | 61  62 | 75  77 | NA | | |
| TIVO-3 | Phase III open-label RCT | Tivozanib  Sorafenib | 173  170 | CC | 2-3 prior systemic  regimens | 62  63 | 72  73 | 19  21 | 62  60 | 18  19 |

*F = Favorable; I = Intermediate; P = Poor; IMDC = International Metastatic Renal-Cell Carcinoma Database Consortium; CC = Clear cell.

**eTable 2. Drug dose and costs**

| Drug | Dose | Route | Unit Price ($) | Cost for 1 model cycle ($, 42 days) |
| --- | --- | --- | --- | --- |
| Lenvatinib | 20mg/d | oral | 18.9851/mg | 15947.484 |
| Pembrolizumab | | | | |
| Lenvatinib+ Pembrolizumab arm | 200mg on day 1 of each 21-day | IV | 50.648/mg | 20259.2 |
| Pembrolizumab+ Axitinib arm | 200mg on day 1 of each 21-day for up to 17.5 cycles | IV | 50.648/mg | 20259.2 |
| Nivolumab | | | | |
| Nivolumab+ Cabozantinib arm | 240 mg every 2 weeks | IV | 28.541/mg | 20549.52 |
| Nivolumab+ Ipilimumab (induction phase) | 3 mg/kg * 70 kg every 3 weeks for 4 doses | IV | 28.541/mg | 23974.44 |
| Nivolumab + Ipilimumab (maintenance phase) | 3 mg/kg * 70 kg every 2 weeks | IV | 28.541/mg | 17980 |
| Nivolumab (Second-line treatment) | 3 mg/kg * 70 kg every 2 weeks | IV | 28.541/mg | 17980 |
| Ipilimumab (induction phase) | 1 mg/kg * 70 kg every 3 weeks for 4 doses | IV | 157.225/mg | 44023 |
| Axitinib | 5mg twice/d | oral | 53.01/mg | 5566.05 |
| Avelumab | 10mg/kg * 70 kg every 2 weeks | IV | 8.5331/mg | 18277.90 |
| Sunitinib | 50 mg/d for 4 weeks followed by 2 weeks off treatment | oral | 12.4616/mg | 17446.24 |
| Cabozantinib | | | | |
| Nivolumab+ Cabozantinib arm | 40 mg/d | oral | 8.1883/mg | 13756.344 |
| Second-line treatment | 60mg/d | oral | 8.1883/mg | 20634.516 |
| Sorafenib | 400mg twice/d | oral | 0.87/mg | 7308 |

**eTable 3. Background mortality rate**

Estimates of background mortality rate for each age are provided in the US life table; Arias E, Heron M, Xu J. United States Life Tables, 2019. Natl Vital Stat Rep. 2019; 68:1-65.

| Age (years) | Background | Age (years) | Background | Age (years) | Background |
| --- | --- | --- | --- | --- | --- |
| 18 | 0.000603 | 54 | 0.007003 | 90 | 0.166829 |
| 19 | 0.000698 | 55 | 0.007607 | 91 | 0.185047 |
| 20 | 0.000795 | 56 | 0.008219 | 92 | 0.204441 |
| 21 | 0.000889 | 57 | 0.008857 | 93 | 0.224919 |
| 22 | 0.000970 | 58 | 0.009542 | 94 | 0.246354 |
| 23 | 0.001424 | 59 | 0.010285 | 95 | 0.26890 |
| 24 | 0.001497 | 60 | 0.011098 | 96 | 0.291442 |
| 25 | 0.001561 | 61 | 0.011952 | 97 | 0.314700 |
| 26 | 0.001624 | 62 | 0.012814 | 98 | 0.338142 |
| 27 | 0.001682 | 63 | 0.013657 | 99 | 0.361537 |
| 28 | 0.001737 | 64 | 0.014502 | 100 | 1 |
| 29 | 0.001792 | 65 | 0.015384 |  |  |
| 30 | 0.001847 | 66 | 0.016444 |  |  |
| 31 | 0.001900 | 67 | 0.017624 |  |  |
| 32 | 0.001952 | 68 | 0.018968 |  |  |
| 33 | 0.002003 | 69 | 0.029586 |  |  |
| 34 | 0.002053 | 70 | 0.022109 |  |  |
| 35 | 0.002111 | 71 | 0.024359 |  |  |
| 36 | 0.002174 | 72 | 0.026347 |  |  |
| 37 | 0.002233 | 73 | 0.028810 |  |  |
| 38 | 0.002285 | 74 | 0.031309 |  |  |
| 39 | 0.002340 | 75 | 0.034486 |  |  |
| 40 | 0.002413 | 76 | 0.038026 |  |  |
| 41 | 0.002516 | 77 | 0.042286 |  |  |
| 42 | 0.002649 | 78 | 0.046547 |  |  |
| 43 | 0.002811 | 79 | 0.051534 |  |  |
| 44 | 0.002999 | 80 | 0.057008 |  |  |
| 45 | 0.003203 | 81 | 0.062923 |  |  |
| 46 | 0.003433 | 82 | 0.069911 |  |  |
| 47 | 0.003709 | 83 | 0.078099 |  |  |
| 48 | 0.004047 | 84 | 0.086754 |  |  |
| 49 | 0.004445 | 85 | 0.096549 |  |  |
| 50 | 0.004874 | 86 | 0.106472 |  |  |
| 51 | 0.005331 | 87 | 0.119677 |  |  |
| 52 | 0.005844 | 88 | 0.134128 |  |  |
| 53 | 0.006408 | 89 | 0.149846 |  |  |

eTable 4. The results of scenario analyses.

| Strategy | Total cost | LY | QALY | ICER |
| --- | --- | --- | --- | --- |
| Scenario 1 | | | | |
| 5 years | | | | |
| Avelumab + Axitinib | 383934.59 | 2.56 | 1.93 | Dominated |
| Nivolumab + Ipilimumab | 292035.84 | 2.66 | 1.99 | Dominated |
| Lenvatinib + Pembrolizumab | 531232.38 | 2.90 | 2.22 | Dominated |
| Sunitinib | 226457.23 | 2.59 | 1.84 | Dominate |
| Pembrolizumab + Axitinib | 266916.70 | 2.72 | 2.06 | 183906.68 |
| Nivolumab + Cabozantinib | 447383.57 | 2.98 | 2.25 | 949825.63 |
| 10 years | | | | |
| Avelumab + Axitinib | 430319.03 | 2.98 | 2.20 | Dominated |
| Nivolumab + Ipilimumab | 305880.59 | 3.11 | 2.28 | Dominated |
| Lenvatinib + Pembrolizumab | 560916.13 | 3.37 | 2.54 | Dominated |
| Sunitinib | 236457.93 | 2.90 | 2.02 | Dominate |
| Pembrolizumab + Axitinib | 274316.23 | 3.19 | 2.38 | 105161.94 |
| Nivolumab + Cabozantinib | 472416.99 | 3.66 | 2.72 | 582649.29 |
| 20 years | | | | |
| Avelumab + Axitinib | 433510.97 | 3.04 | 2.33 | Dominated |
| Nivolumab + Ipilimumab | 308711.31 | 3.20 | 2.40 | Dominated |
| Lenvatinib + Pembrolizumab | 565971.26 | 3.42 | 2.62 | Dominated |
| Sunitinib | 236575.54 | 2.95 | 2.11 | Dominate |
| Pembrolizumab + Axitinib | 271005.09 | 3.34 | 2.52 | 83974.51 |
| Nivolumab + Cabozantinib | 481316.11 | 3.88 | 2.95 | 489095.40 |
| Scenario 2 | | | | |
| 10% patents switch to BSC | | | | |
| Avelumab + Axitinib | 429619.89 | 3.05 | 2.31 | Dominated |
| Nivolumab + Ipilimumab | 297001.34 | 3.18 | 2.39 | Dominated |
| Lenvatinib + Pembrolizumab | 553849.10 | 3.38 | 2.60 | Dominated |
| Sunitinib | 230135.81 | 2.89 | 2.07 | Dominate |
| Pembrolizumab + Axitinib | 265428.99 | 3.28 | 2.50 | 82077.16 |
| Nivolumab + Cabozantinib | 486760.57 | 3.64 | 2.76 | 851275.31 |
| 30% patents switch to BSC | | | | |
| Avelumab + Axitinib | 410713.40 | 2.93 | 2.19 | Dominated |
| Nivolumab + Ipilimumab | 278153.32 | 3.06 | 2.25 | Dominated |
| Lenvatinib + Pembrolizumab | 533362.37 | 3.29 | 2.44 | Dominated |
| Sunitinib | 210638.87 | 2.74 | 1.91 | Dominate |
| Pembrolizumab + Axitinib | 247524.64 | 3.19 | 2.37 | 80186.46 |
| Nivolumab + Cabozantinib | 466098.35 | 3.44 | 2.53 | 1366085.69 |
| Scenario 3 | | | | |
| Change second-line treatment sequence to nivolumab | | | | |
| Avelumab + Axitinib | 421502.11 | 2.99 | 2.21 | Dominated |
| Nivolumab + Ipilimumab | 512913.50 | 3.07 | 2.27 | Dominated |
| Lenvatinib + Pembrolizumab | 549596.21 | 3.36 | 2.50 | Dominated |
| Sunitinib | 239124.70 | 2.40 | 1.63 | Dominate |
| Pembrolizumab + Axitinib | 261303.72 | 3.24 | 2.41 | 30382.22 |
| Nivolumab + Cabozantinib | 512913.50 | 3.51 | 2.62 | 1198141.81 |
| Change second-line treatment sequence to axitinib | | | | |
| Avelumab + Axitinib | 426299.12 | 3.22 | 2.42 | Dominated |
| Nivolumab + Ipilimumab | 473138.00 | 3.71 | 2.79 | Dominated |
| Nivolumab + Cabozantinib | 491614.35 | 3.85 | 2.90 | Dominated |
| Sunitinib | 209482.24 | 3.40 | 2.48 | Dominate |
| Pembrolizumab + Axitinib | 259626.48 | 3.50 | 2.59 | 455856.73 |
| Lenvatinib + Pembrolizumab | 538847.99 | 4.06 | 3.11 | 536964.44 |
| Scenario 4 | | | | |
| Adjust nivolumab 75% of its original price in the first-line setting. | | | | |
| Sunitinib | 243437.11 | 2.96 | 2.11 | - |
| Nivolumab + Cabozantinib | 424916.80 | 3.75 | 2.81 | 259256.70 |
| Adjust nivolumab 50% of its original price in the first-line setting. | | | | |
| Sunitinib | 238911.16 | 2.96 | 2.10 | - |
| Nivolumab + Cabozantinib | 358062.81 | 3.75 | 2.79 | 172683.55 |
| Adjust nivolumab 25% of its original price in the first-line setting. | | | | |
| Sunitinib | 235597.22 | 2.92 | 2.09 | - |
| Nivolumab + Cabozantinib | 296201.96 | 3.69 | 2.82 | 83020.19 |

eFigure 1. Model Structure.


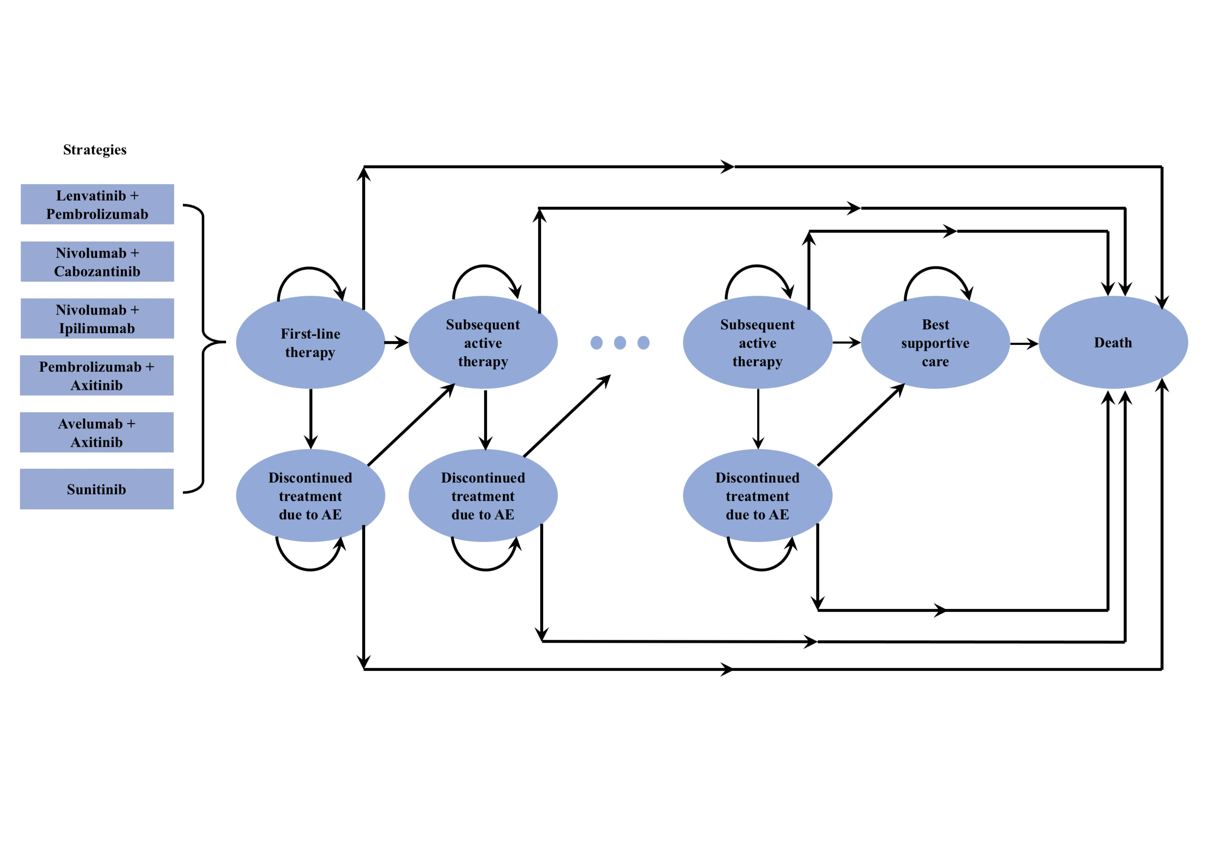


eFigure 2. The Cost-Effective Frontier of 6 Different Competing Strategies.


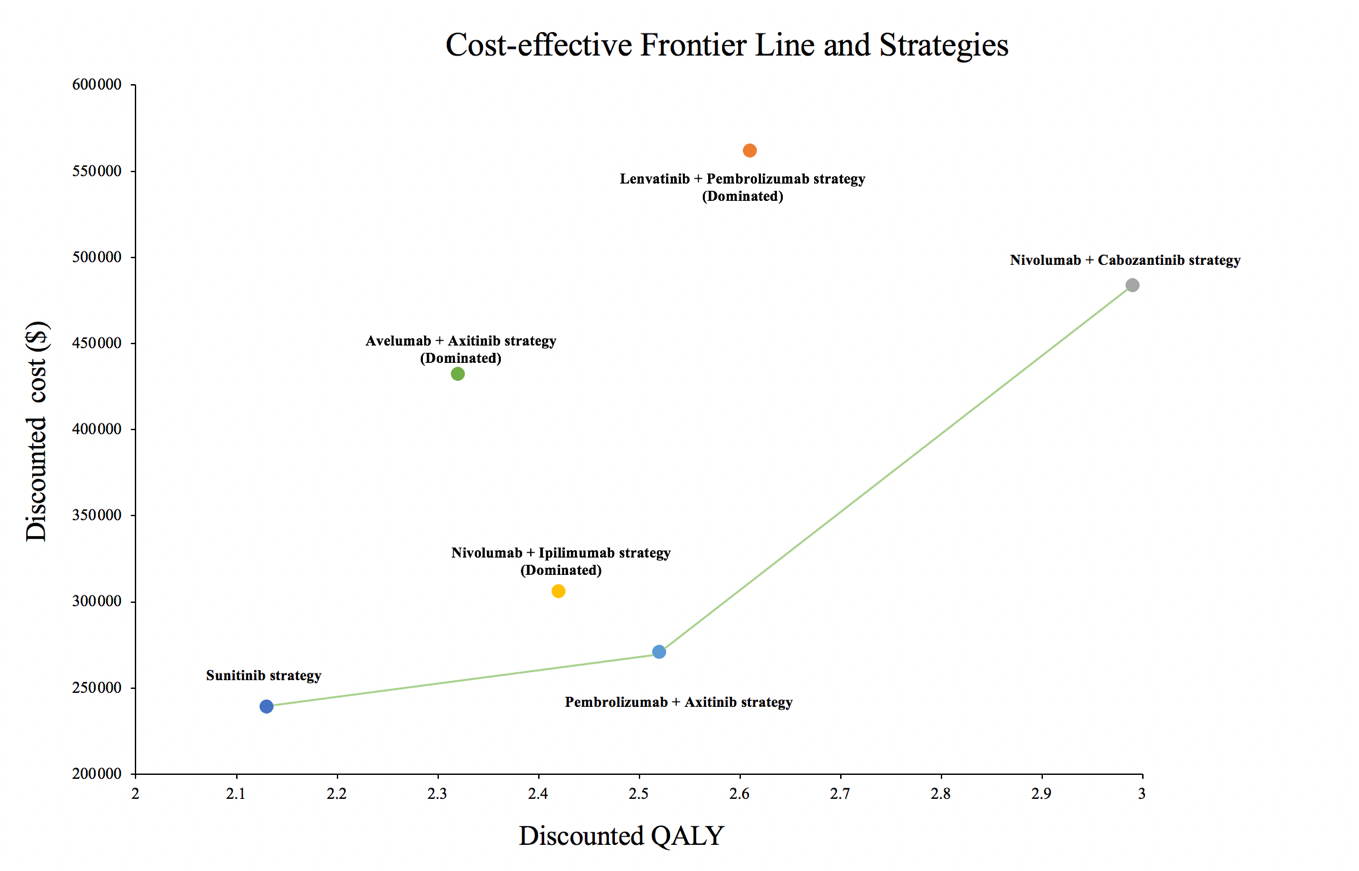


eFigure 3. Tornado Diagrams Showing the Effect of Lower and Upper Values of Each Parameter on the ICERs of the Pembrolizumab-plus-Axitinib Versus Sunitinib Strategy.


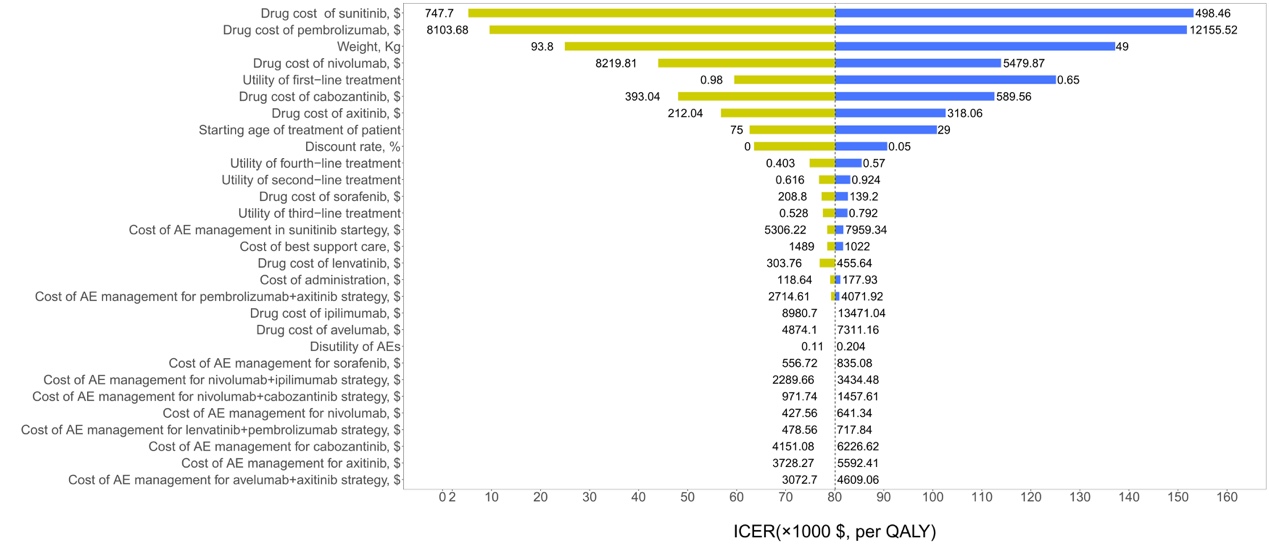


eFigure 4. Tornado Diagrams Showing the Effect of Lower and Upper Values of Each Parameter on the ICERs of the Nivolumab-plus-Cabozantinib Versus Sunitinib Strategy.


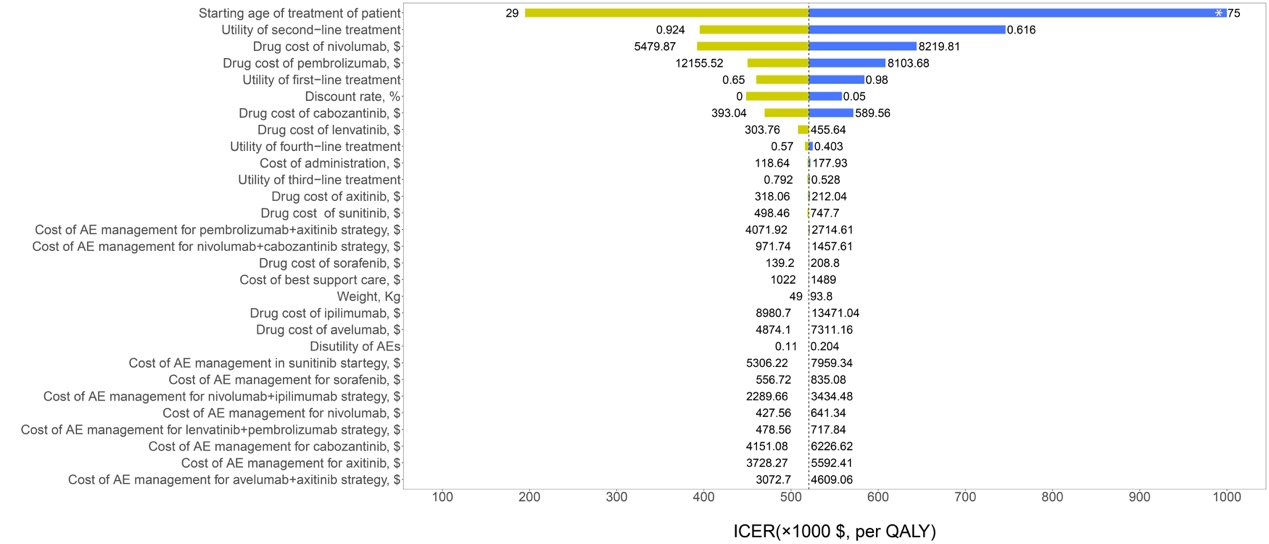


eFigure 5: Parametric Distributions for First-Line Treatment.

*KM = Kaplan-Meier; LEN+PEM = Lenvatinib + Pembrolizumab; NIV+CABO =Nivolumab + Cabozantinib; NIV+IPI = Nivolumab + Ipilimumab; PEM+AXI = Pembrolizumab + Axitinib; AVE+AXI = Avelumab + Axitinib


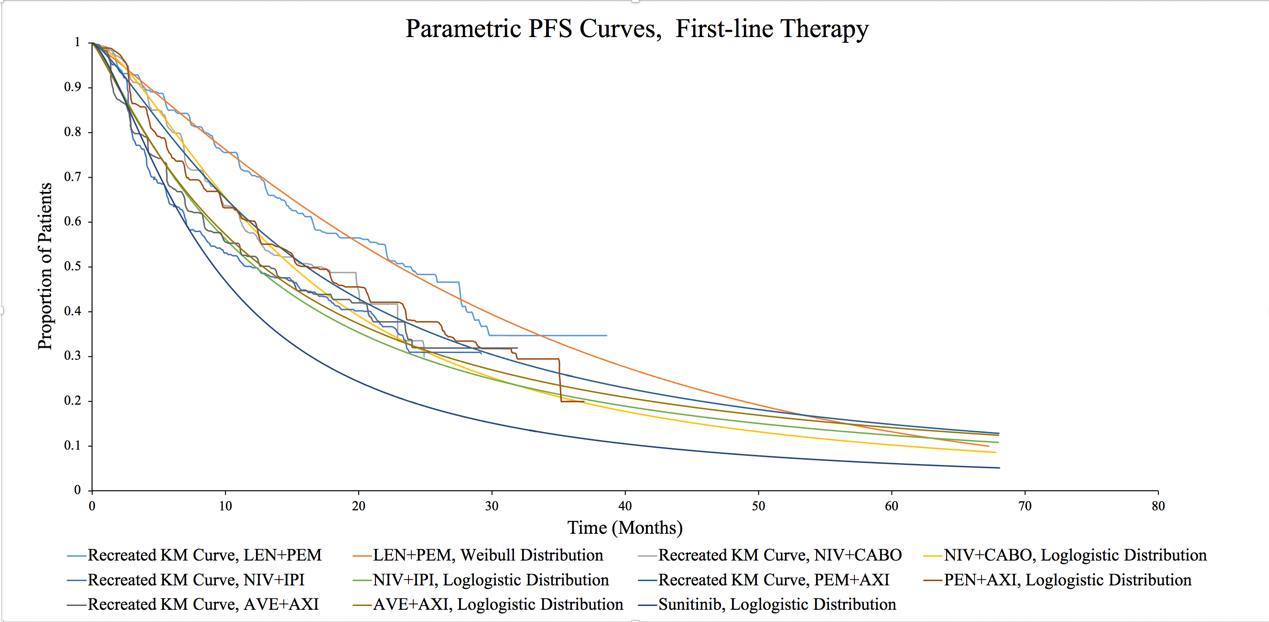


eFigure 6: Parametric Distributions for Second-Line Treatment. KM = Kaplan-Meier.


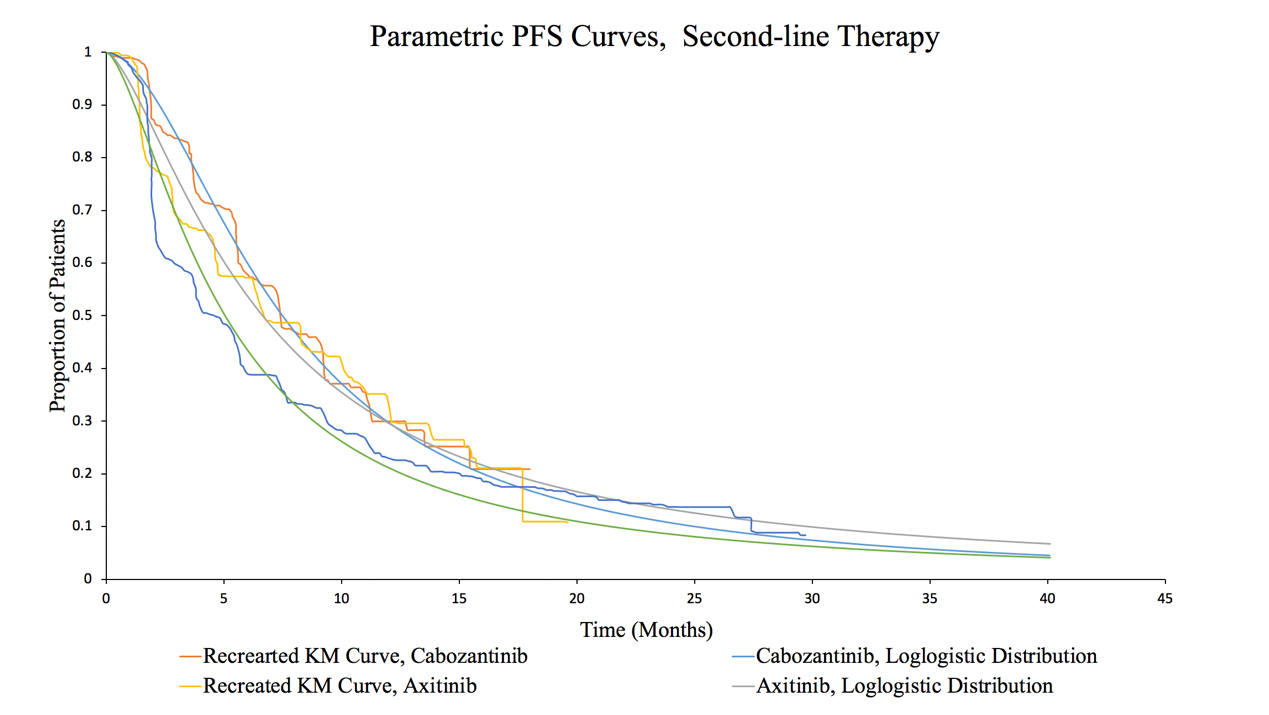


eFigure 7: Parametric Distributions for Third-Line Treatment. KM = Kaplan-Meier.


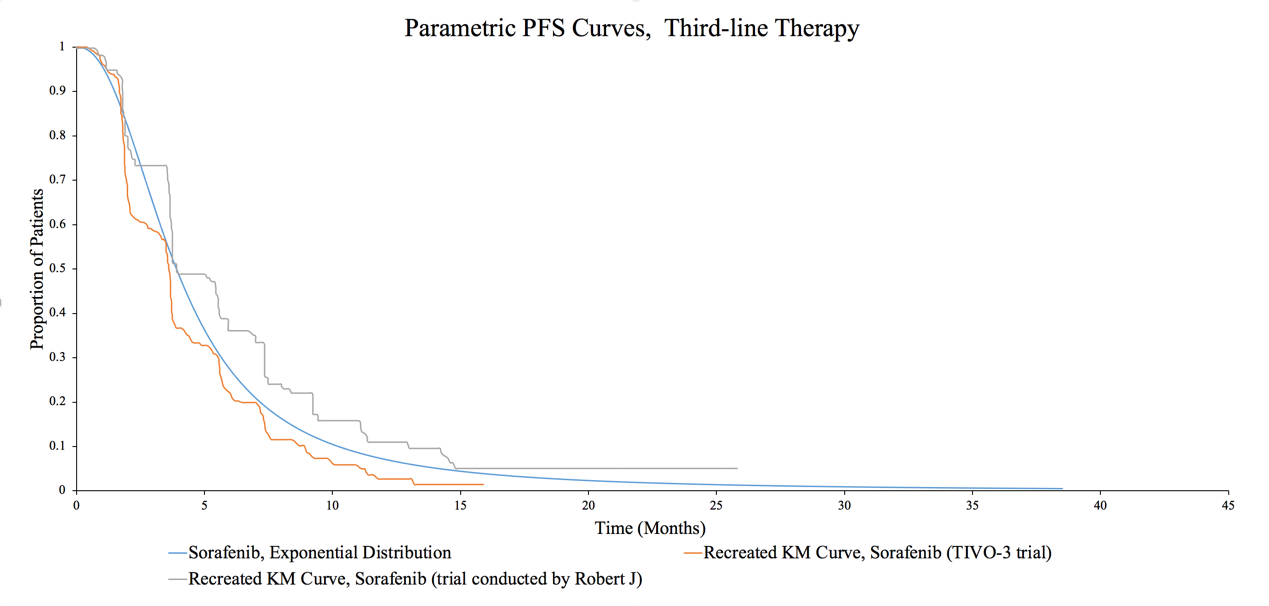


eFigure 8: Parametric Distributions for Best Support Care State.

*BSC = Best support care.


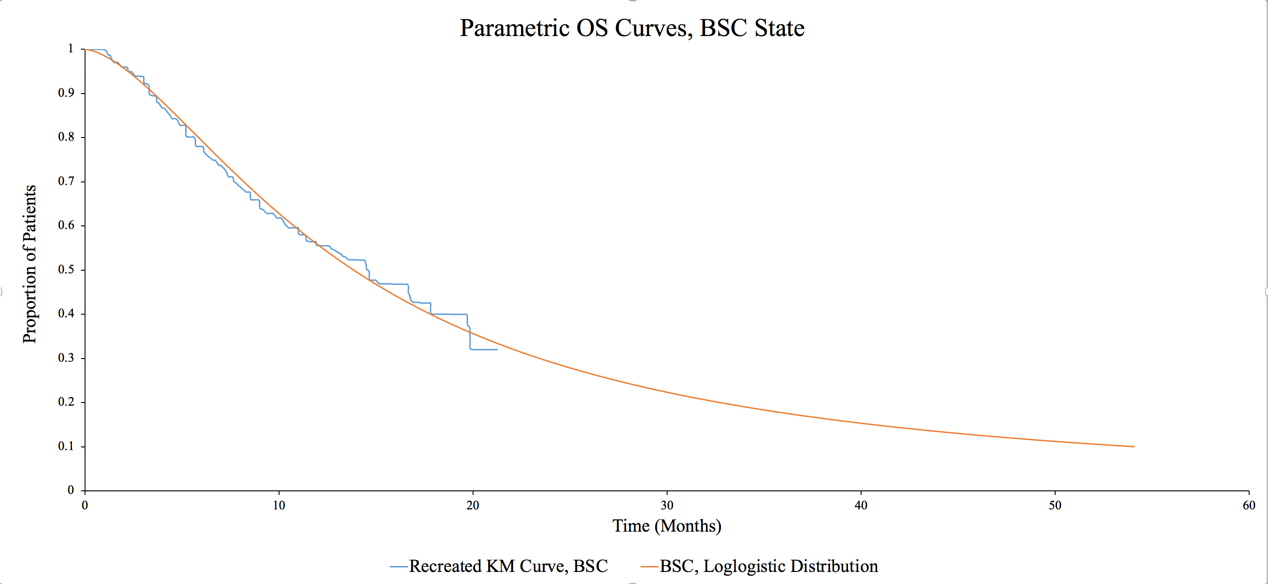

Supplement: Supplementary file 1 [file DataSheet1.DOCX]
